# Utility of Pelvic MRI and Tumor Markers HE4 and CA125 to Predict Depth of Myometrial Invasion and Cervical Involvement in Endometrial Cancer

**Published:** 2015-11

**Authors:** Narges Zamani, Mitra Modares Gilani, Fateme Zamani, M. Hossein Zamani

**Affiliations:** 1 Department of Gynecology and Obstetrics, Tehran University of Medical Sciences, Tehran, Iran; 2 Department of Radiology, Tehran University of Medical Sciences, Tehran, Iran; 3 Tehran University of Medical Sciences, Tehran, Iran

**Keywords:** Endometrial Carcinoma, CA125, HE4, MRI, Surgical Staging

## Abstract

**Objective:** The purpose of this pilot study was to determine whether the MRI and biomarkers human epididymis protein 4 (HE_4_) and CA_125_ correlate with depth of myometrial invasion, histologic grade, cervical involvement and nodal metastases in patients with endometrioid adenocarcinoma of the uterus.

**Materials and methods:** This was a prospective, observational study in women with biopsy-proven endometrial adenocarcinoma of the uterus. Preoperative pelvic MRI was performed and concentration of HE_4_ and CA_125 _were assessed before surgery. All surgical specimens were reviewed by a single expert pathologist. The results were compared with the final histopathology report of surgical staging.

**Results:** Included were a total of 68 women with endometrioid adenocarcinoma of the uterus, most (76%) with stage I disease. Levels of serum HE_4_ greater than 140PM and CA_125_ greater than 35 ku/L were observed in 12 (17%) and 26 (38.2%) of patients respectively whose greater proportion were cases with deep myometrial invasion and high grade tumor. In the evaluation of deep tumoral invasion (> 50%) of the myometrium sensitivity, specificity, and diagnostic accuracy of MRI were 68.9%, 94.8% and 83.8% respectively. For lymph node involvement these values were 50%, 95.1% and 91.1% respectively and for cervical stromal involvement were 64.3%, 98.1% and 91.1% respectively.

**Conclusion:** Higher stage, deep myometrial invasion, and lymph node or cervical stromal involvement increase diagnostic accuracy of MRI. Higher levels of HE_4_ and CA_125_ were observed in patients with deep myometrial invasion and higher grade of tumor.

## Introduction

Endometrial cancer is the most common gynecologic cancer (1). And is also known to be the fourth commonest cancer overall and the seventh leading cause of death by cancer in North America (2). It is well recognized the prognosis for this disease is intimately related to the surgical stage. 

In 1988 the international federation of Gynecology and Obstetrics (FIGO) system changed staging for endometrial cancer from clinical to surgical (3). In addition, the American College of Obstetricians and Gynecologists currently advocates systematic surgical staging, including pelvic washings, bilateral pelvic and para-aortic lymphadenectomy, and a complete resection of all disease as the primary treatment for the most women with endometrial cancer (4).

However lymphadenectomy can be associated with trouble some sequelae, including symptomatic lymphocyst, lymphedema, deep vein thrombosis, neurologic injury, vascular injury, and the need for blood transfusion (5, 6).

In stage I disease, lymphadenectomy has been reported to result in moderate to severe complications in 13% of patients (7).

Because of the potential morbidity associated with Lymphadenectomy, some centers argue that the indicate for Lymph node dissection should be based on a consideration of risk factors for nodal metastasis, such as tumor grade histology and extent of disease beyond the endometrium (8).

Investigators have shown that in low-risk endometrial disease, lymph node metastasis occurs in 5% of patients and the risk for lymph node metastasis if associated with histologic grade, invasion into the capillary- lymphatic space and extent of myometrial invasion (9).

A preoperative test that is sensitive and specific for endometrial cancer and correlated with risk factors associated with nodal metastases in early disease could be useful in identifying cases that would benefit from lymphadenectomy and excluding those that would not. In addition, combining these tests with prognostic factors that can be determined in the preoperative period such as tumor grade, histology and patient age may more accurately help determine which patients would benefit from lymphadenectomy.

Currently there is no single reliable test or algorithm to determine preoperatively the likelihood of lymph node metastases in women with endometrial cancer. 

CA_125_ is a cell surface antigen expressed by different types of tumor cells. Measurement of serum CA_125_ is commonly used in evaluation and follow up process of patients with ovarian cancer. However these is little published information regarding preoperative CA_125_ levels in patients with endometrial cancer and the relationship of these level to stage of disease (10). 

Human epididymis protein 4 (HE_4_), a novel tumor marker, circulates in the bloodstream and is overexpressed in patients with serous and endometrioid epithelial ovarian carcinomas and uterine cancers (11, 12).

Magnetic Resonance Imaging (MRI) is a noninvasive diagnostic method for preoperative staging of endometrial cancer. A gynecologic oncology surgeon could then consider that information in addition to other clinical factors when planning for surgery and in making the decision to perform a lymphadenectomy.

Approximately 75% of patients with endometrial cancer are diagnosed at an early stage, when the prognosis is usually excellent. However 25% of patients are diagnosed at more advanced stages (2). In these cases, through surgical staging becomes very important to guide the need for adjuvant treatment. MRI and the serum CA_125_, HE_4_ levels is thus of growing interest in the preoperative investigation of women with endometrial cancer. Because of its potential to predict the presence of more advanced stage disease. Patients suspected of having more advanced stage disease, could then potentially be triaged and referred preoperatively to gynecologic oncologists for complete surgical staging.

## Materials and methods

This was a prospective institutional review board approved (ref number: 9237/941) and an observational pilot study which was conducted at Valie-asr Hospital, Tehran, Iran. The diagnosis of endometrial cancer was done by endometrial biopsy or functional curettage. Preoperative investigation including a complete history and physical examination, a chest x-ray abdominal and pelvic ultrasound, MRI, routine lab test and serum HE_4_ and CA_125_ levels was done for each patient, and most of these women with endometrioid adenocarcinoma then underwent total abdominal hysterectomy, bilateral salpingo-oophorectomy with bilateral pelvic and periaortic lymph node dissections. 

Depth of myometrial invasion, FIGO histologic grade, cervical stromal involvement and presence or absence of nodal metastases were recorded.

Patients with medical problems which could potentially elevate CA_125_ HE_4_ levels were excluded.

Fish exact-chi-square kappa index testing and multivariate logistic regression were performed to examine the effects of clinicopathological factors on serum CA_125_, HE_4_ levels. Value of p < 0.05 was considered statistically significant.

## Results

A total of 90 women with endometrial cancer enter into the trial, 80 of whom were diagnosed with an endometrioid adenocarcinoma of the uterus were evaluable.

**Table 1:** Relation between serum CA125 level and histopathological prognostic factors

	CA125 < 35 ku/l [n (%)]	CA125 ≥ 35 ku/l [n (%)]	p
**FICO**			**0.002**
** I**	**38 (73.1%)**	**14 (26.9%)**	
** II**	**3 (33.3%)**	**6 (66.7%)**	
** III**	**1 (14.3%)**	**6 (85.7%)**	
**Myometrial Invasion **			**0.0001**
** No invasion**	**1 (100%)**	**0(0)**	
** < 50%**	**32 (84.3%)**	**6 (15.7%)**	
** ≥ 50%**	**9 (14.3%)**	**6 (68.9%)**	
**Grade**			**0.001**
** I**	**23 (82.2%)**	**5 (17.8%)**	
** II**	**19 (31.1%)**	**16 (45.7%)**	
** III**	**0 (0)**	**5 (100%)**	
**Node Involvement**			**0.002**
** No**	**42 (66.7%)**	**20 (33.3%)**	
** Yes**	**0 (0)**	**6 (100%)**	
**Cervical Stromal Involvement**			**0.006**
** No**	**38 (70.4%)**	**16 (29.6%)**	
** Yes**	**4 (28.6%)**	**10 (71.4%)**	

Twelve patients were excluded because these was no tumor marker recorded in their medical chart. Twenty five patients were (36.8%) under 50 years, thirty seven patients (54.4%) were between 50 and 70 years and six patients (8.8%) were upper 70 years. Fifty two patients (76.5) had stage I nine patients (13.2%) had stage II and seven patients (10.3%) had stage III, IV disease. With regard to serum CA_125_ levels 42 patients (61.8%) had values < 35ku/L and 26patients (38.2%) had levels ≥35 ku/L.

Fourteen of out 52 patients (26.9 %) with stage I had CA125 level ≥ 35 ku/l compared with six (66.7 %) of the nine patients with stage II and six (85.7%) of the seven patients with stage III (p < 0.002) (Tables 1 and 2).

The association between poor histopathological prognostic factors and CA_125_ level is shown in tables 1 and 2. A significant increase in serum CA_125_ level was noted in patients with grade 3 tumors, deep myometrial invasion, cervical stromal involvement, and nodal metastases (Table 3).

Among the group of patients with early stage disease a significant increase in serum CA_125_ was noted in patients with deep myometrial invasion.

With regard to serum HE_4_ levels, 56 patients (82.4%) had values < 140 PM and 12 patients (17.6%) had levels ≥ 140 PM. Five out of 52 patients (9.6%) in stage I had HE4 level ≥ 140PM. compared with 3 patients (33.3%) with stage II and 4 patients (57.1%) with stage III disease (p < 0.003) (Tables 3 and 4).

**Table 2:** Relation between serum CA125 level and histopathological prognostic factors in early stage carcinoma

	CA125 ≥ 35ku/l n (%)
**Myometrial invasion**	
** < 50%**	**6 (23.1%)**
** ≥ 50%**	**20 (76.9%)**
**Grade**	
** I**	**5 (19.2%)**
** II**	**16 (61.6%)**
** III**	**5 (19.2%)**
**Node Involvement**	
** No**	**26 (76.9%)**
** Yes**	**6 (23.1%)**
**Cervical stromal Involvement **	
** NO**	**16 (61.6%)**
** Yes**	**10 (38.4%)**

The association between poor histopathological prognostic factors and HE_4_ level is shown in table 3, 4.

A significant increase in serum HE_4_ level was noted in patients with grade III tumors, deep myometrial invasion, cervical stromal involvement and nodal metastasis (Table 3, 4)

Among the group of patients with early stage disease a significant increase in serum HE_4_ was noted in patients with deep myometrial invasion (Tables 3 and 4).

**Table 3:** Relation between serum (HE4) level and histopathologieah prognostic factor

	HE4 < 140PM [n (%)]	HE4 ≥ 140PM [n (%)]	p
**FICO**			**0.003**
** I**	**47 (90.4%)**	**5 (9.6%)**	
** II**	**6 (66.7%)**	**3 (33.3%)**	
** III**	**3 (42.9%)**	**4 (57.1%)**	
**Myometrial invasion**			**0.001**
** No**	**1 (100%)**	**0 (0)**	
** < 50%**	**37 (97.4%)**	**1 (2.6%)**	
** ≥ 50%**	**18 (62.1%)**	**11 (37.9%)**	
**Grade**			**0.035**
** I**	**27 (96.5%)**	**1 (3.5%)**	
** II**	**25 (71.5%)**	**10 (28.5%)**	
** III**	**4 (80%)**	**1 (20%)**	
**Node involvement **			**0.007**
** No**	**54 (85.2%)**	**8 (14.8%)**	
** Yes**	**2 (33.4%)**	**4 (66.6%)**	
**Cervical stromal Involvement**			
** No**	**48 (88.9%)**	**6 (11.1%)**	
** Yes**	**8 (57.2%)**	**6 (42.8%)**	

In the surgicopathological report in one patient (1.5%) had no involvement of the myometrium, 38 patients (55.9%) had less than 50% and 29 patients (42.6%) had equal or greater than 50% involvement of myometrium. From 19 patients who did not have any myometrial invasion in the MRI report, had 17 patients lower than 50% and one patients more than 50% myometrial invasion Eight out of 27 patients with less than 50% myometrial involvement in MRI ultimately had more than 50% myometrial involvment Twenty out of 22 patient with an MRI report of more than 50% myometrial invasion had the same finding in final pathology reports.

In the evaluation of deep myometrial invasion (more than 50%) the sensitivity, specificity, diagnostic accuracy, positive and negative predictive values were 68.9%, 94.8%, 83.8%, 90.9%, 80.4% respectively (Table 5).

According to the correlation of cervical invasion to MRI and the final pathology report 53 out of 58 patients without cervical stromal involvement in MRI not have any cervical invasion in the surgico pathological report, 5 patients had stromal involvement. 9 out of 10 patients with cervical stromal involvement in MRI have stromal invasion in the surgico pathological report. For cervical stromal involvement the sensitivity, specificity, diagnostic accuracy, positive and negative predictive values were 64.3%, 98.1%, 91.1%, 90%, 91.4% (Table 5).

**Table 4:** Relation between serum HE4 level and histopathological prognostic factors in early stage carcinoma

	HE4 > 140PM n (%)
**Myometrial Invasion**	
** < 50%**	**1 (8.3%)**
** > 50%**	**11 (91.7%)**
**Grade**	
** I**	**1 (8.3%)**
** II**	**10 (83.4%)**
** III**	**1 (8.3%)**
**Node Invasion**	
** NO**	**8 (66.7%)**
** Yes**	**4 (33.3%)**
**Cervical stromal Involvement**	
** No**	**6 (50%)**
** Yes**	**6 (50%)**

**Table 5:** Diagnostic performance regarding detection of myometrial and cervical invasion

	TP	FP	FN	TN	Sensitivity %	Specificity %	Accuracy %	PPV*	NPV**
**FICO**									
** I**	**34**	**2**	**18**	**14**	**65.4%**	**78.5%**	**70.5%**	**94.4%**	
** II**	**5**	**3**	**4**	**56**	**55.5%**	**94.9%**	**89.7%**	**62.5%**	
** III**	**3**	**3**	**4**	**58**	**42.8%**	**95.1%**	**89.7%**	**50%**	**93.5%**
**Myometrial Invasion**									
** No **	**1**	**18**	**0**	**50**	**100%**	**73.5%**	**75%**	**5.2%**	**100%**
** < 50%**	**19**	**8**	**19**	**22**	**50%**	**73.3%**	**60%**	**70.3%**	**53.6%**
** ≥ 50%**	**20**	**2**	**9**	**37**	**68.9%**	**94.8%**	**83.8%**	**90.9%**	**80.4%**
**Node involvement**									
** No**	**59**	**3**	**3**	**3**	**95.1%**	**50%**	**91.1%**	**95.1%**	**50%**
** Yes**	**3**	**3**	**3**	**59**	**50%**	**95.1%**	**91.1%**	**50%**	**95.1%**
**Cervical stromal involvement**									
** No**	**53**	**5**	**1**	**9**	**98.1%**	**64.3%**	**91.1%**	**91.4%**	**90%**
** Yes**	**9**	**1**	**5**	**53**	**64.3%**	**98.1%**	**91.1%**	**90%**	**91.4%**

* Positive predictive value;

** Negative predictive value

## Discussion

Endometrial cancer is the most common gynecologic cancer (1) and it is well recognized that the prognosis for this disease is intimately related to the surgical stage. Because of the potential morbidity associated with lymphadenectomy some centers argue that the indicate for lymph node dissection should be based on a consideration of risk factors for nodal metastasis such as tumor grade histology and extent of disease beyond the endometrium.

A preoperative test that is sensitive and specific for endometrial cancer and correlated with risk factors with nodal metastasis in early disease could be useful in identifying cases that would benefit from lymphadenectomy and excluding those that would not MRI tumor markers (CA125– HE4) are non-invasive diagnostic methods for preoperative staging of endometrial cancer.

Our data clearly show a significant increase in serum CA_125_ level in patients with grade 3 tumors, deep myometrial invasion, cervical stromal involvement and noda metastasis (Tables 1 and 2).

Our data compare favorably with previously published data. In 2010 Sebastianellia et al. associated assay of preoperative serum CA_125_ level is a very simple test to detect patients with more advanced stage endometrial adenocarcinoma (13). Its routine use could help triage high risk patients preoperatively. In 2002 Hsieh et al. associated elevated preoperative CA_125_ levels with an increased risk of metastatic disease at the time of surgery and could be a good indication for performing full pelvic lymphadenectomy (14). Following a review of 92 patients, Chung et al. reported in 2006 that an elevated CA_125_ level was a good predictor of lymph node metastasis in women with endometrial cancer (15). 

In our data clearly show a significant increase in serum HE_4_ level in patients with grade 3 tumors, deep myometrial invasion, cervical stromal involvement and node metastasis. Our data compare favorably with previously published data. In 2011 Moore et al. associated HE_4_ may be a useful marker preoperatively in the clinical decision process for determining the need for lymph node dissection in women with endometrial cancer (16). 

Although modalities such as CT scan and MRI are not a part of FIGO staging for endometrial carcinoma.

MRI can provide valuable data to estimate staging and choose the best treatment planning (17).

The treatment of choice for endometrial cancer is surgery based on staging cervical stromal involvement and the depth of myometrial invasion, the type of surgery can differ from a simple hysterectomy without lymphadenectomy or only a lymph node sampling to radical surgery with systematic lymphadenectomy (18). Moreover with MRI we can decide about conservation management for patients who want fertility preservation.

The overall accuracy and specificity for cervical invasion ranged from 45-98% and 87-100% (19). It has been proven that the depth of myometrial invasion has a direct relation with lymph node involvement and prognosis (6-7 fold). Avoidance of unnecessary lymphadenectomy is important in low risk patients based on the depth of myometrial invasion, grading before surgery.

In our data the evaluation of deep tumoral invasion of the myometrium (> 50%) sensitivity, specificity and diagnostic accuracy of MRI were 8.9%, 94.8% and 83.8% (Table 5).

In 2008 Savelli et al. associated 84%, 71% and 88% to be sensitivity, specificity and diagnostic accuracy of MRI (19). In 2007 Vasconcelosa et al. showed sensitivity and specificity to be 89% and 100% (20). 

In our data for cervical stromal involvement sensitivity, specificity and diagnostic accuracy were 67.3%, 98.1% and 91.1%. In 2008 Savelli et al these values were 79%, 87% and 84% (19).

## Conclusion

Measurement of serum CA_125_, HE_4_ are a simple test to perform in the preoperative evaluation of women with endometrial cancer. Higher stage, deep myometrial invasion and lymph node or cervical stromal involvement increase diagnostic accuracy of MRI. Higher levels of HE_4_ and CA_125_ were observed in patients with deep myometrial invasion and higher grade of tumor. Theirs routine use could help triage high risk patients for referral to a gynecologic oncologist, for full staging.
